# Intestinal Microbiota and Gene Expression Reveal Similarity and Dissimilarity Between Immune-Mediated Colitis and Ulcerative Colitis

**DOI:** 10.3389/fonc.2021.763468

**Published:** 2021-10-27

**Authors:** Kazuko Sakai, Toshiharu Sakurai, Marco A. De Velasco, Tomoyuki Nagai, Takaaki Chikugo, Kazuomi Ueshima, Yurie Kura, Takayuki Takahama, Hidetoshi Hayashi, Kazuhiko Nakagawa, Masatoshi Kudo, Kazuto Nishio

**Affiliations:** ^1^ Department of Genome Biology, Faculty of Medicine, Kindai University, Osaka, Japan; ^2^ Department of Gastroenterology and Hepatology, Faculty of Medicine, Kindai University, Osaka, Japan; ^3^ Department of Diagnostic Pathology, Faculty of Medicine, Kindai University, Osaka, Japan; ^4^ Department of Medical Oncology, Faculty of Medicine, Kindai University, Osaka, Japan

**Keywords:** immune-related adverse event, ulcerative colitis, immune-checkpoint inhibitor, pathway enrichment analysis, gene expression, microbiota

## Abstract

Immune checkpoint inhibitors (ICIs) have become the standard of care for several cancers. However, ICI therapy has also been associated with various immune-related adverse events (irAEs). Clinical manifestations of immune-related colitis resemble those of inflammatory bowel diseases such as ulcerative colitis (UC). The composition of the bowel microflora is thought to influence the development of inflammatory bowel disease and irAE colitis. We profiled the gene expressions and microbe compositions of colonic mucosa from patients with solid cancers receiving anti-PD-L1 antibody treatment; we then compared the expression profiles associated with irAE colitis with those associated with UC. The pathway enrichment analysis revealed functional similarities between inflamed regions of irAE colitis and UC. The common enriched pathways included leukocyte extravasation and immune responses, whereas non-inflamed mucosa from patients with irAE colitis was distinct from patients with UC and was characterized by the recruitment of immune cells. A similarity between the microbiota profiles was also identified. A decreased abundance of *Bacteroides* species was observed in inflamed regions from both irAE colitis and UC based on a microbiota composition analysis of 16S rDNA sequencing. Pathways associated with molecule transport systems, including fatty acids, were enriched in inflamed and non-inflamed irAE colitis and inflamed UC, similar to Piphillin-inferred KEGG pathways. While UC is characterized by local regions of inflammation, ICI treatment extends to non-inflammatory regions of the colonial mucosa where immune cells are reconstituted. This analysis of the similarity and heterogeneity of irAE colitis and UC provides important information for the management of irAE colitis.

## Introduction

Monoclonal antibodies targeting immune checkpoint cytotoxic T lymphocyte antigen-4 (CTLA-4), programmed death receptor-1 (PD-1) and programmed death ligand 1 (PD-L1), referred to as immune checkpoint inhibitors (ICIs), have become a new standard of care for several cancers including lung cancer and melanoma (1–3) as well as renal cell carcinoma (4). ICIs can be distinguished from other targeted therapies and chemotherapies in that they enhance anti-tumor T-cell activity, whilst traditional antineoplastic agents exert direct cytotoxic effects (1–3). Therefore, although long-term survival is expected in some patients, ICIs could lead to a new class of immune-related adverse events (irAEs), of which gastrointestinal (GI) irAEs are among the most frequent and severe (5, 6). In their clinical presentation and endoscopic findings, irAE colitis resemble ulcerative colitis (UC) (7, 8). In our previous study, we have reported on the association between responsiveness to ICI and the gut microbiota (9). However, the functional relationship between ICI-induced irAE and the gut microbiota remains unclear.

IrAEs are common and diverse, varying in incidence, timing, and severity. Rapid diversification and clonal expansion of T cells, which can take place early in ICI treatment (10, 11), may contribute to irAE pathogenesis. Epitope spreading allows diversified T cells to attack not only more targets on the tumor but also normal tissues, leading to irAEs (12). Additionally, B cells interact intimately with T cells to produce antibodies with high reactivity against non-self and low reactivity against self. ICIs, in causing iatrogenic T cell hyperactivation, additionally have been demonstrated to drive the peripheral accumulation of activated B-cell subpopulations and antibody-producing plasmablasts, which have been correlated with irAE development and severity (13). Expanded, activated B cells may contribute both directly and indirectly to irAE pathogenesis through mechanisms that may include cytokine production, further antigen presentation to T cells, and increased secretion and diversification of autoreactive antibodies. The innate immune system comprises a wide variety of cells, including dendritic cells, natural killer (NK) cells, macrophages/monocytes, neutrophils, and innate lymphoid cells (ILCs). Together, they contribute to irAE pathogenesis likely both in cooperation with and independent of adaptive immune cells. Moreover, studies have reported associations of irAEs with neutrophil-mediated inflammatory response and formation of neutrophil extracellular traps, as well as high levels of circulating eosinophils (14, 15). Changes in the global immune response are facilitated and reflected by the milieu of circulating cytokines, which includes anti-inflammatory cytokines (e.g., IL-10, TGF-β), pro-inflammatory innate immune cytokines (e.g., IL-1, IL-6), and pro-inflammatory adaptive immune cytokines (e.g., IFN-γ, IL-17) (16). ICIs may tip the balance of this milieu toward inflammation and autoimmunity (17). IL-17, for example, is a cytokine secreted by helper T cells (specifically TH17 cells) that acts to suppress regulatory T cells (Tregs) (17, 18). While TH17 cells have been described to play a pathogenic role in autoimmune disorders (18), Tregs have immunosuppressive functions important in maintaining peripheral tolerance (17, 18). ICIs may sway this balance in favor of an increased TH17 cell response with robust IL-17 secretion and away from Treg-mediated self-tolerance, which has been hypothesized to contribute to ICI-induced colitis (19). In the signaling pathways, IFN-γ activates JAK-STAT signaling to induce PD-L1 expression on tumor cells, allowing them to evade peripheral immune surveillance. JAK-STAT activation by IFNs is also important for T cell activation and differentiation (20), and aberrant signaling along various IFN-JAK-STAT axes is known to contribute to the pathogenesis of many autoimmune diseases (21) and irAE such as myocarditis due to anti-PD-1 therapy (22). It is therefore plausible that the possible mechanisms driving irAEs include (i) activation of cytotoxic T cells; (ii) activation of B cells and increased autoantibody production; (iii) direct molecular mimicry and off-target toxicity; (iv) activation of intracellular signaling and pro-inflammatory cytokine production; and (v) environmental modifiers of immune system activation, including composition of the host gut microbiome. These mechanisms may help identify predictive biomarkers and targeted treatment strategies.

To gain additional insights and to shed additional light into the link between ICI therapy and irAEs, we herein investigated the microbiota and gene expressions in inflamed and uninflamed mucosae of irAE colitis and UC patients. A functional enrichment analysis of the whole transcriptome and 16S rDNA sequencing data obtained using an Ingenuity^®^ Pathway Analysis (IPA) and Piphillin prediction (23) allowed us to compare the functional attributes of the microbial communities in the studied environments. To the best of our knowledge, this is the first study to examine the similarities in gut bacterial communities and gene signatures between irAE colitis and UC.

## Methods

### Patients and Controls

Eighteen patients with immune checkpoint inhibitor-induced (irAE) colitis, 9 with ulcerative colitis (UC), and three healthy individuals were included in this study. The irAEs were defined as adverse events (AEs) with a potential immunologic basis that required more frequent monitoring and potential intervention with immune suppression or endocrine therapy (24). Asymptomatic and transient abnormalities of laboratory findings were not regarded as irAEs (i.e., asymptomatic subtle thyroid stimulating hormone (TSH) or aspartate aminotransferase (AST)/alanine aminotransferase (ALT) elevation spontaneously resolving without any interventions etc.). Concurrent irAE was defined multi-organ irAE during a same treatment cycle. AEs occurring after initiation of next treatment were not collected. All AEs were graded according to Common Terminology Criteria for Adverse Events (CTCAE) version 4 (25). In patients with gastrointestinal irAEs, clinical symptoms such as diarrhea and bloody stool occurred at a median of 3 months (1-10 months) after ICI initiation. UC patients were diagnosed based on their clinical histories and endoscopic and histological findings. In these patients, intestinal biopsy tissue from an inflamed region (active) and a non-inflamed region (inactive) were collected. The endoscopic activity of colitis was assessed according to the Mayo endoscopic subscore (0: normal, 1: mild, 2: moderate, 3: severe). Evaluation of the degree of inflammation was performed according to the Geboes score of ulcerative colitis. The tissue sites evaluated were those with the highest degree of endoscopic and histological inflammation in the multiple tissues collected. Endoscopic and histological evaluations were performed by a physician who is both a gastrointestinal endoscopist and a gastroenterologist. Non-inflamed mucosae were defined as mucosae that were endoscopically not inflamed; biopsies were taken from the right colon (Mayo endoscopic subscore 0) or the ileum. For controls, biopsies were taken from uninflamed mucosae from 3 non-IBD patients and feces samples were collected from 3 healthy individuals. All samples were immediately frozen and stored at −80°C.

### Ethical Approval

This study was conducted in compliance with the Helsinki Declaration and the Ethical Guidelines for Medical and Health Research Involving Human Subjects by the Japanese government. This study was approved by the ethical committee of the Kindai University Faculty of Medicine (28-224).

### DNA/RNA Isolation

DNA and RNA extraction from intestinal biopsy tissue samples were performed using an AllPrep DNA/RNA Mini Kit (Catalog #80204, Qiagen, Valencia, CA). The quality and quantity of the nucleic acid were verified using a NanoDrop 2000 device, PicoGreen dsDNA Reagent (Catalog #P7581), and RiboGreen RNA reagent (Catalog #R11491) from Thermo Scientific (Wilmington, DE).

### Whole-Transcriptome Analysis

For the whole transcriptome analysis, we used the AmpliSeq Transcriptome Human Gene Expression Kit (Catalog #A26325, Thermo Fisher Scientific). The RNA extracted from tissue samples were reverse-transcribed using the SuperScript VILO cDNA Synthesis kit (Catalog #11754050, Thermo Fisher Scientific), followed by multiplexed PCR amplification, end repair, and barcoded-adaptors ligation according to the manufacturer**’**s instructions.

Pooled libraries were subjected to the Ion Chef System (Thermo Fisher Scientific) for template preparation. Libraries were then loaded onto an Ion 550 chip and sequenced with the Ion S5 sequencing system. The Ion Torrent Suite v5.10 software (Thermo Fisher Scientific) was used for base calling, alignment to the human reference genome (hg19), and quality control. Raw reads were then analyzed automatically using the AmpliSeqRNA plugin to generate gene-level expression values for all 20,802 RefSeq human genes.

### 16S rDNA Sequencing and Data Analysis

16S rDNA sequencing was performed using the V3-V4 16S rRNA region for paired-end sequencing on the Illumina MiSeq platform or the V2, V3, V4, V6, V7, V8, and V9 16S rRNA region for single-end sequencing on the Thermo Fisher Scientific Ion S5 platform. 16S rDNA sequencing using the Illumina MiSeq platform was performed on inactive intestinal biopsy tissue samples as previously described (9). Briefly, the V3-V4 16S rRNA region was amplified, followed by index PCR for adaptor ligation. The pooled library was sequenced as paired-end 300-bp reads using MiSeq Reagent Kit V3 (Illumina). 16S rDNA sequencing using the Thermo Fisher Scientific Ion S5 platform was performed on active intestinal biopsy tissue samples. The 16S rDNA library was prepared with the Ion 16S Metagenomics Kit (Thermo Fisher Scientific), according to the manufacturer’s instructions. For library preparation, the V2, V3, V4, V6, V7, V8, and V9 16S rRNA region was amplified, followed by end repair and barcoded-adaptors ligation using the Ion Plus Fragment Library Kit (Thermo Fisher Scientific). The pooled library was sequenced as single-end 400-bp reads using the Ion S5 sequencing kit (Thermo Fisher Scientific).

The FASTQ files were analyzed using the CLC Genomics Workbench version 12.0 (Qiagen) and the Microbial Genomics Module (Qiagen). Sequence reads were clustered into operational taxonomic units (OTUs) with a 99% identify threshold against the Greengenes database, version 13.8. A set of sequences representing OTUs were analyzed using Calypso (version 8.84) (26) OTU abundance was normalized with cumulative-sum scaling (CSS) and log2 transformation. Samples with a total read count <1000 were filtered from subsequent analyses.

### Pathway Enrichment Analysis

Pathway analysis of the gene expression data from human tissues was conducted using IPA (Version: 51963913) (Qiagen). Genes with a fold change greater than 4 (the absolute value of a log2 fold change greater than 2) and an adjusted p value less than 0.05 were considered as being differentially expressed and were subjected to IPA. The IPA analysis provided a Z-score that predicted the direction of the change in the dataset and a p value from a right-hand Fisher exact test. Positive Z-scores predicted activation, and negative Z-scores predicted inactivation of the enriched pathway.

Prediction of the functional profiles from the metagenomic 16S rDNA sequencing analysis was performed using the online Piphillin server (KEGG version October 2018) (23).

### Statistical Analyses

Statistical analyses were performed using JMP (version 14.0; SAS Institute, Cary, NC), GraphPad Prism software (Version 8; GraphPad Software Inc., La Jolla, CA), and XLSTAT for Microsoft Excel (Addinsoft SARL; Paris, France) for statistical analysis. The Pearson correlation coefficient was used for quantifying linear correlation. The Mann-Whitney U-test was used to compare two groups. *P* values of less than 0.05 were considered statistically significant.

## Results

### Patient Characteristics

In total, 18 patients with irAE colitis who had diarrhea and underwent a colonoscopy were included. Clinical symptoms such as diarrhea occurred at a median of 3 months (1-10 months) after ICI initiation. Two patients were refractory to corticosteroids and were treated with tumor necrosis factor (TNF) blockade using infliximab: one patient had a perforation, and the other patient underwent an ileostomy because of unsuccessful medical treatment including cyclosporin and cytapheresis. Patients had cancer of the lung (11), stomach (2), kidney (3), ovary (1), and unknown origin (1) (**Table 1**). UC patients were significantly younger than those with irAE colitis. IrAE colitis and UC were prominent in male and female patients, respectively, but not statistically significant because of small sample size. On the other hand, patients with moderate or severe colitis were frequently observed in ulcerative colitis group compared with irAE colitis group, although it was not significant. There was no significant difference in grade score between irAE colitis and UC, although there was a trend toward higher grade in UC. Most patients with irAE colitis were able to tolerate subsequent ICI therapy. However, all the patients who restarted ICI therapy relapsed after ICI restart (median time, 5 months; range, 1–17 months). Concurrent irAEs (lung 2, hypothalamus 1, skin 1, brain 1) developed in 5 patients with irAE colitis.

**Table 1:** Patient characteristics.

		Immune checkpoint inhibitor-induced colitis (irAE) n = 18	Ulcerative colitis (UC)n = 9	*P* value
Age, years	Median (range)	66.5 (43 to 79)	23.0 (14 to 49)	<0.001
Gender	Male	13 (72.2%)	3 (33.3%)	1.000
	Female	5 (27.8%)	6 (66.6%)	
Geboes score grade	Grade 1	3 (16.7%)	1 (11.1%)	0.426
	Grade 2	9 (50.0%)	2 (22.2%)	
	Grade 3	1 (5.6%)	1 (11.1%)	
	Grade 4	5 (27.8%)	5 (55.6%)	
Colitis activity	Mild	9 (50.0%)	1 (11.1%)	0.059
	Moderate	7 (38.9%)	4 (44.4%)	
	Severe	2 (11.1%)	4 (44.4%)	
Cancer types	Lung	11 (61.1%)	–	–
	Stomach	2 (11.1%)	–	
	Kidney	3 (16.7%)	–	
	Ovary	1 (5.6%)	–	
	Unknown	1 (5.6%)	–	
Availability of inactive tissue	Yes	14 (77.8%)	9 (100%)	–
	No	4 (22.2%)	0 (0%)	
Availability of active tissue	Yes	15 (83.3%)	9 (100%)	–
	No	3 (16.7%)	0 (0%)	

*P values were based on the Fisher’s exact test for categorical data and the Mann-Whitney U test for continuous data.

### Similarity of Gene Expression Profiles Between irAE and UC

Data from a whole transcriptome analysis of RNA extracted from colon biopsy samples was used to select differentially expressed genes (fold change >2 and p <0.05, ANOVA) in active and inactive regions of irAE and UC relative to normal controls. Based on these conditions, a total of 511, 1,698, 2,906 and 4,090 genes were obtained from non-inflamed (inactive) regions of irAE (I-irAE, n=14) and UC (I-UC, n=9) and from inflamed (active) regions of irAE colitis (A-irAE, n=15) and UC (A-UC, n=9), respectively.

**Figure 1:**
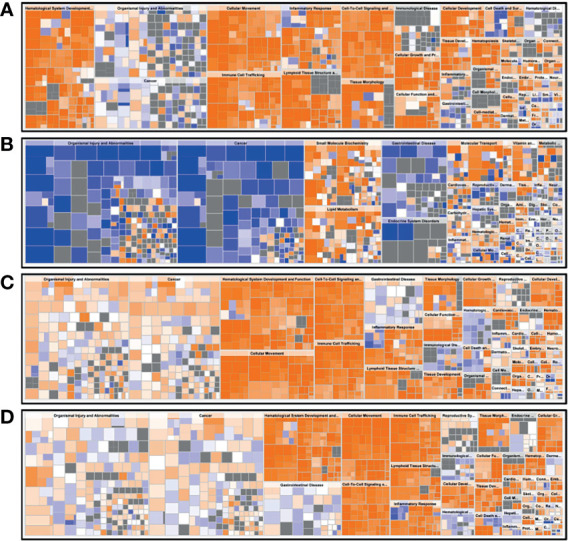
Biological trends for I-irAE, I-UC, A-irAE, and A-UC regions compared with normal samples. The size and color of each tile reflect the *p* value (−log) and the z-score, which reflect the overall predicted activation state (<0: inhibited, >0: activated), respectively, compared with the normal sample. The intensity scale of the z-score ranges from dark blue (low value) to dark orange (high value). **(A)** Featured canonical pathways of inactive irAE (*n* = 14) and normal control (*n* = 3). **(B)** Featured canonical pathways of inactive UC (*n* = 9) and normal control (n = 3). **(C)** Featured canonical pathways of active irAE (*n* = 15) and normal control (*n* = 3). **(D)** Featured canonical pathways of active UC (*n* = 9) and normal control (*n* = 3).

**Figure 2:**
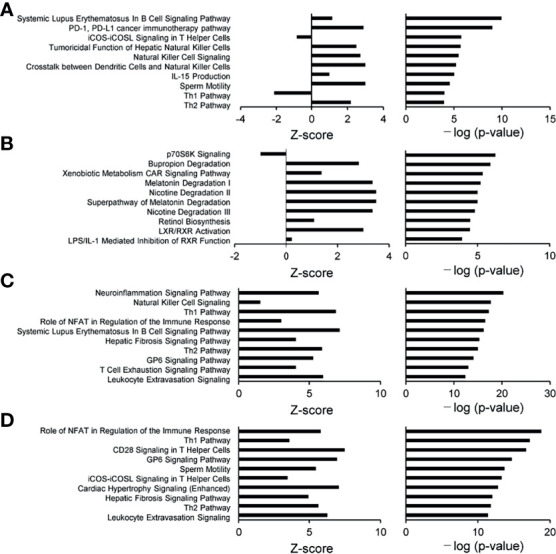
Diagrams of the top 10 canonical pathways in an enrichment analysis. The bar plots show the z-score, which reflects the overall predicted activation state (<0: inhibited, >0: activated) (left), and the *p* value (−log) (right). **(A)** Ten most statistically significant canonical pathways of inactive irAE and a normal control. **(B)** Ten most statistically significant canonical pathways of inactive UC and a normal control. **(C)** Ten most statistically significant canonical pathways of active irAE and a normal control. **(D)** Ten most statistically significant canonical pathways of active UC and a normal control.

A functional characterization analysis was performed using IPA to identify canonical pathways enriched in each group. **Figure 1** shows the biological trends of the I-irAE, I-UC, A-irAE, and A-UC regions compared with normal samples using the Z-score and p-value. The I-irAE and I-UC regions showed different tones, while the A-irAE and A-UC regions were visually similar. Of the 712 canonical pathways, 14, 13, 108, and 101 pathways were significantly enriched in the I-irAE, I-UC, A-irAE, and A-UC regions, respectively, when compared with normal samples. In inactive regions of irAE and UC, pathways related to immune cells and responses, such as B cell, helper T cell, and natural killer cells, were enriched in I-irAE (**Figure 2A**). Furthermore, the Th2 pathway was enriched and inversely correlated with the Th1 pathway, suggesting a Th2-dominant milieu. However, P70 S6 kinase (p70S6K) signaling and metabolic pathways, particularly those associated with substance degradation, were enriched in I-UC; notably, no overlap occurred between pathways enriched in I-irAE and those enriched in I-UC (**Figure 2B**). Conversely, immune-related pathways such as NK cells, Th1, Th2 and the immune response as well as neuro-inflammation pathways were enriched in the A-irAE cohort. Remarkably, six of the top ten canonical pathways enriched in A-irAE were also enriched in A-UC and included those involved in immune response and leukocyte extravasation (**Figures 2C, D**). Overall, these results indicated a functional similarity between A-irAE and A-UC, but not between non-inflamed regions, based on the gene expression profiles.

To further examine the functional similarity between irAE and UC, we performed a Pearson correlation analysis using the Z-scores of the enrichment scores from the canonical pathways that were analyzed (**Figure S1**). Generally, moderately positive correlations were observed between I-irAE and I-UC (r=0.5482) and between A-irAE and I-irAE (r=0.7112) (**Figures S1A, B**). However, a strong positive correlation was observed between the A-irAE and A-UC (r =0.9581) regions (**Figure S1C**), whereas none were present between the inactive and active regions of UC (**Figure S1D**). Overall, these results show some similarity between inflamed and non-inflamed regions in irAE, but more notably, our findings revealed a strong similarity between inflamed mucosal regions of irAE and UC.

To investigate the mechanisms that could explain similarities between inflammatory irAE and inflammatory UC, we analyzed pathways common to A-UC and A-irAE. Among the 239 genes comprising six pathways common to A-irAE and A-UC, we extracted the top 100 genes that differed from normal samples using the signal-to-noise ratio, followed by unsupervised hierarchically clustered based one minus Pearson correlation (**Figure S2**). The clusters that were upregulated in both A-irAE and A-UC compared to normal samples included *ICAM1*, *ITGB2*, *CD28*, *CD40*, *CD80*, *CD86*, and *CD4*, which are upstream regulatory molecules related to inflammation. The gene expression of those genes was found to be significantly increased compared to normal samples. These molecules are suggested to contribute common molecular events in inflamed UC as well as inflamed irAE.

We further examined the differences in gene expressions between A-irAE and A-UC directly and extracted the featured pathways. The most activated pathway observed in A-UC was “crosstalk between dendritic cells and natural killer cells” pathway (**Figure S3A**). This suggests an innate immune mechanism were specifically activated in A-UC. Furthermore, tumor necrosis factor (TNF) was selected as the most predictive upstream regulatory molecules in A-UC (**Figure S3B**), which could be related with the activation of innate immunity.

### Similarity of Microbial Composition Between irAE and UC

**Figure 3:**
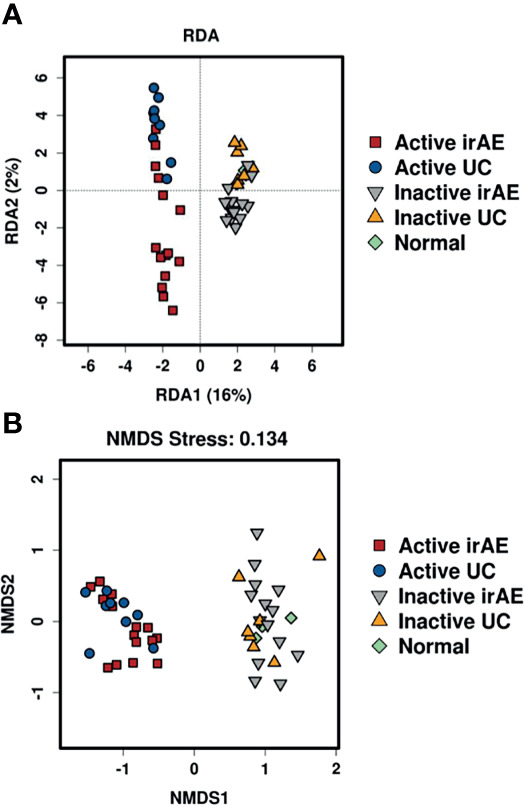
Comparison of biodiversity indices between different groups of tissue microbiota. **(A)** Redundancy analysis (RDA) plot showing the relationship of inflammation and disease status. Plot at operational taxonomic units (OTUs) level. The contribution rate of each component is shown in parentheses. **(B)** Non-metric multidimensional scaling (NMDS) plot showing the relationship of inflammation and disease status. Plot at operational taxonomic units (OTUs) level. The NMDS stress value was 0.134, which is considered to be statistically reliable due to it is less than 0.2.

To characterize the intestinal microbial composition, colonial mucosa samples were submitted for microbiota profiling using 16S rDNA sequencing. The cleaned sequences were clustered into OTUs with a sequence identity threshold of 99%. A redundancy analysis (RDA) showed inflammation (RDA1) as the primary factor influencing taxa (**Figure 3A**). A non-metric multidimensional scaling (NMDS) analysis also showed that the groups could be classified as active and inactive components (**Figure 3B**).

### Hierarchical Clustering and Relative Abundance of Taxa

**Figure 4:**
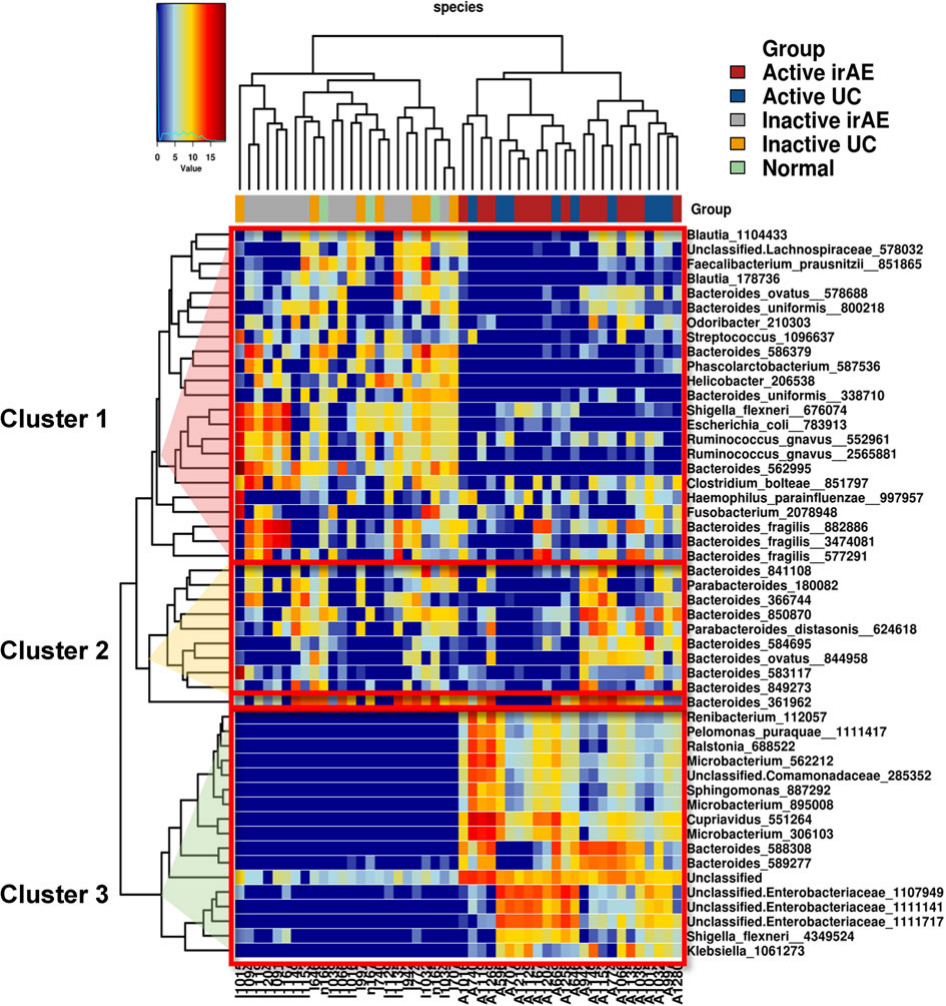
Heatmap of unsupervised hierarchical clustering of tissue microbiota composition at species level based on the Bray-Curtis distance metric. The bacteria that are shown represent the 50 most abundant taxa across all fraction samples. Taxa abundance is shown according to color ranging from red (highly abundant) to blue (rare or absent) and the values of taxa correspond to the heatmap scale on top of the main heatmap.

Significant differences in the relative abundances of bacterium species were analyzed. A heatmap of the top 50 contributing bacterial species visualized three different clusters consisting of taxa with a low (Cluster 1), no difference (Cluster 2), and high (Cluster 3) normalized abundance in inflamed regions (compared with non-inflamed and normal samples) (**Figure 4**). Cluster 1 contained abundant taxa in I-irAE, I-UC, and normal samples. The results for normal tissue were clustered together with those for non-inflamed samples. Cluster 3 was characterized by enriched abundant taxa in the inflamed samples (A-irAE and A-UC). No distinct clusters of irAE and UC were formed independently. The normalized abundance of 50 genera and the significant differences between sample groups are listed in **Table S1**. Compared with the normalized abundance between inflamed *vs.* non-inflamed samples, significant differences were observed in 16/23, 1/10 and 17/17 of the taxa species in Clusters 1, 2, and 3, respectively (**Table S1**). Among the 16 taxa with significant differences between the inflamed *vs.* non-inflamed groups in Cluster 1, significant decrease in the normalized abundances of 11 species in A-irAE and 11 species in A-UC were observed when compared with normal mucosa. A decreased normalized abundance of the Cluster 1 species was commonly observed in inflamed (but not in non-inflamed) irAE and UC mucosa. *Bacteroides* was the most abundant taxa in the top 50 species. *Bacteroides*_586379 and _562995 were significantly absent in inflamed irAE and UC. In Cluster 3, *Enterobacteria* and other species were enriched in inflamed regions, as reported previously (27, 28). The enrichment of *Microbacterium* in inflamed regions was detected as has been previously reported for IBD patients (29). Together, the abundance compositions of the top 50 taxa at the species level were similar between the inflamed regions of irAE and UC and the non-inflamed regions of irAE and UC, respectively.

### Inferred Microbial Function

**Figure 5:**
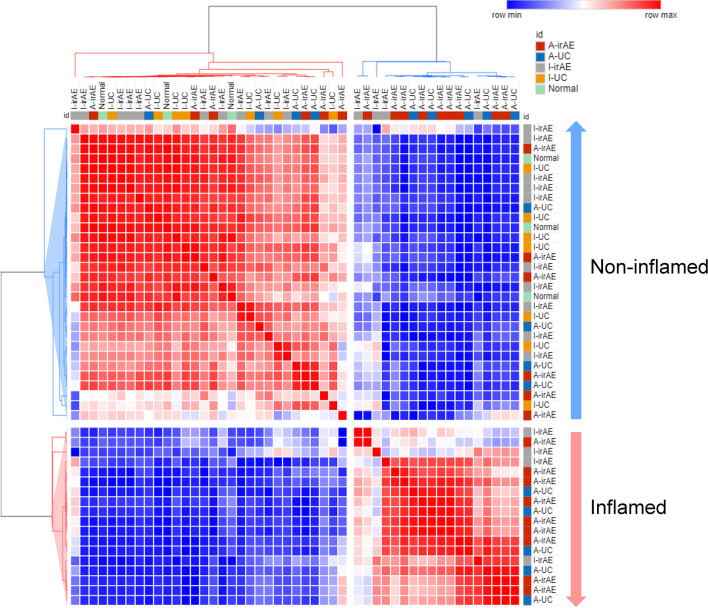
Correlation heatmap of relative abundance of 50 most abundant inferred KEGG orthology pathways selected by Piphillin in I-irAE (*n* = 14), I-UC (*n* = 9), A-irAE (*n* = 15), A-UC (*n* = 9), and normal samples (*n* = 3) using Pearson’s correlation metric. Values were scaled to z-score and colors correspond to correlation, red = high correlation and blue = low correlation. Dendrograms represent average distance. Two main clusters were identified, and samples characterized by inflamed (A-irAE and A-UC) and non-inflamed (I-irAE, I-UC, and normal) status were correlated accordingly.

Furthermore, the potential consequences for community functioning were assessed based on 16S rDNA data using Piphillin. Of the estimated 342 metagenomic KEGG pathway functions, the 50 most abundant pathways were selected by Piphillin. **Figure 5** shows the correlation heatmap for the relative abundances of the 50 most abundant pathways in I-irAE, I-UC, A-irAE, A-UC, and normal samples. In the functional pathway analysis, inflamed regions tended to be classified as different from non-inflamed regions. The top 50 abundant pathways of each group were compared with the normal pathways. A significant difference in the abundance of these 50 pathways between inflamed and non-inflamed groups *vs.* normal regions was seen, and 23 pathways exhibited significantly different abundance between non-inflamed irAE *vs.* normal regions (p<0.05) (**Table S2**). No differences in abundant pathways were observed between non-inflamed UC *vs.* normal regions. This finding indicates functional similarities between irAE colitis and UC microbiota in inflamed irAE regions. Furthermore, 5 pathways were selected based on a large fold-change (Log2 ratio>2) in abundance between A-irAE and normal regions. Five pathways have been characterized by Piphillin as being associated with intestinal flora: butanoate and fatty acid metabolism, quorum sensing ATP binding cassette transporters, and two-component systems. This evidence suggests that this dysbiosis may affect the transport systems of substances from the flora to the mucosa and mucosal defense.

A comparison of the predicted functional pathways suggests the similarity of the microbiota profile between inflamed regions of irAE and UC. No common significant pathways in inflamed and non-inflamed UC were seen, while 23 of the 50 pathways differed significantly between inflamed and non-inflamed irAE regions.

## Discussion

This study investigated the similarities and differences in irAE, UC, and normal samples by comparing the gene expression profiles of colonial mucosal tissues and microbiota composition. Furthermore, the statuses of inflamed and non-inflamed regions in the same patients were compared to determine whether the results of a detailed examination of the local mucosa would reflect the pathological condition.

The importance of the local flora component has been discussed in IBD (30), but few reports have discussed local differences in irAE colitis. The present study showed similar and distinct profiles of mucosal gene expression and microbiota composition. Clinically, similarities and differences between irAE colitis and IBD, including UC, have been reported. IrAE exerts a similar phenotype, histology, and serological characteristics to those of IBD (Crohn’s disease and UC), but with a more aggressive course of disease with acute inflammation (31–33). On the other hand, the similarities in the gene expression profiles in regions of irAE and UC have not been clarified until this study.

Gene expression profiling and microbial composition analyses of colonial mucosa, showed similarities between irAE and UC, suggesting that analyses of the local mucosa and microbial composition might be important for understanding the pathogenesis of irAE and IBD.

The strong correlation between the Z-scores for the inflamed regions of irAE and UC suggests a large degree of similarity. A correlation analysis of the Z-scores for the whole transcriptomes was able to quantify the degree of similarity and was found to be useful for evaluating similarity. These results were consistent with the clinical feature that UC is a local inflammatory lesion and that no changes are seen in non-inflammatory areas (34, 35). The canonical pathways of enrichment analysis also demonstrated a similarity between inflamed irAE and UC regions, but not between non-inflamed irAE and UC regions. Immune cell reconstitution may have occurred in non-inflamed regions of irAE, but not of UC.

Gender differences in IBD have been reported for epidemiology and other factors. In IBD, gender-specific differences have been reported for Crohn’s disease (CD), but not ulcerative colitis (UC). In Europe and the United States, CD prevalence appears to be higher in females than in males (36, 37), while in Asia the opposite has been observed (38, 39). These results have potential clinical implications. Sex-associated differences exists in immune systems. Immune checkpoint inhibitor monotherapy-associated hepatitis is frequently observed in males in Japan (40). On the other hand, no clear evidence of gender difference of irAE colitis has not been reported. A randomized controlled trial demonstrated that of 1,011 patients who began treatment with pembrolizumab (anti-PD1) therapy or placebo, 622 (61.5%) were men and 389 (38.5%) were women; 386 patients (38.2%) were aged 50 to 64 years, 377 (37.3%) were younger than 50 years, and 248 (24.5%) were 65 years and older (41). A meta-analysis using The Cancer Genome Atlas (TCGA) omics data also shows and validated that minimal sex-associated differences in irAEs including irAEs colitis among cancer patients who received immune checkpoint inhibitor therapy (42). Thus, it may be unnecessary to consider gender effects for irAE management in clinical practice.

In this study, we evaluated the transcriptome of irAE colitis in the patients treated with mainly anti-PD-1 blockade. Compared to other immune checkpoint inhibitors, irAE colitis induced by anti-CTLA-4 are frequent, potentially severe and resemble IBD, whereas those induced by PD-1 blockade seem to be less frequent and clinically more diverse. Three recent systematic literature reviews and meta-analyses of published studies have assessed the risks of diarrhea and colitis in patients treated with anti-PD-1 and/or anti-CTLA-4 (6). The incidence of colitis was 0.7%–1.6% for anti-PD-1, 5.7%–9.1% for anti-CTLA-4% and 13.6% for the combination of both therapies. It may be necessary to consider the impact of therapeutic targets on irAE colitis management in clinical practice.

Unsupervised microbial abundance clustering also showed distinct taxa compositions between inflamed and non-inflamed regions. This result is consistent with previous reports of flora taxonomy in IBD patients (43) suggesting that inflamed regions are likely to be reflected in the flora of IBD. Andoh et al. reported that the bacterial composition during the remission (non-inflamed) phases of UC, but not Crohn**’**s disease, resembled that of normal mucosa (31). While we did not investigate Crohn**’**s disease in the present cohort, we are interested in exploring the similarities between irAE and Crohn**’**s disease in a future study.

The top 5 enriched pathways shared by inflamed irAE, UC, and non-inflamed irAE regions were selected during an inferred microbial function analysis performed using Piphillin. Reportedly, pathways related to butanoate and fatty acid metabolism are closely related to IBD colitis (32). A decrease in anaerobic bacteria represented by *Clostridium* and *Fusobacterium* leads to a decrease in butyric acid production, abnormal mucosal repair, decreased anti-inflammatory activity, and the defective induction of regulatory T cells. These events may lead to the onset of IBD (33, 44). However, *Clostridium* and *Fusobacterium* were not abundant in the presently reported cohort. Other contributing bacteria should be present. The concentration of quorum-sensing molecule secreted by bacteria is known to control bacterial growth and to regulate immunity (45, 46). The activation of the two-component system through an increase in the abundance of *Enterobacteriaceae* is involved in the pathogenesis of colitis (47). An association between the two-component system and *Enterobacteriaceae* may exist, since various *Enterobacteriaceae* were selected as the flora in Cluster 3. Bacteria secretes proteins into the extracellular environment and mediates interactions between bacteria and eukaryotic hosts (48). Type I secretion systems are very similar to ATP-binding cassette (ABC) transporters in many Gram-negative bacteria, and ABC transporters that secrete small molecules out of the cell (49). Colitis was preceded by altered gut bacterial composition, suggesting that the deletion of ATP-binding cassette B1 (Abcb1) led to fundamental changes in host-microbiota interactions in a mouse model (50). Another critical role of Abcb1 was suggested by the finding that Abcb1 expression identified a subpopulation of pro-inflammatory Th17 cells that were resistant to treatment with glucocorticoids (50). The cytokine-mediated downregulation of the major human efflux transporter ABCB1 in inflamed intestinal tissues in UC patients is presumably dependent on disease activity, with a possible contribution from interleukin-8 (51). A decrease in sigmoidal ABCB1 expression in UC was associated with disease activity (52). Taken together, the present results suggest that enriched pathways related to bacterial molecule transport systems, including fatty acids, are commonly observed in inflamed and non-inflamed irAE and inflamed UC, based on a functional pathway analysis of taxa. We speculated that dysbiosis was related to the onset of inflammation commonly occurring in irAE and UC. In conclusion, ICI treatment extends to the non-inflammatory region of the colonial mucosa, where immune cells are reconstituted, whereas UC consists of local inflammatory lesions. However, this study is limited by its retrospective design and small sample size, thus, future studies examining larger sample sizes are needed to confirm the findings of this study.

## Data Availability Statement

Raw 16S rRNA gene amplicon sequences are be deposited to DNA Data Bank of Japan/Sequence Read Archive (DDBJ/DRA) under the accession number DRA012351, DRA012819, and DRA012820.

## Ethics Statement

The studies involving human participants were reviewed and approved by the ethical committee of the Kindai University Faculty of Medicine (28–224). The patients/participants provided their written informed consent to participate in this study.

## Author Contributions

KS contributed experimental design, data collection, data acquisition, analysis and interpretation of the data, writing the first draft of the paper. TS contributed experimental design, data collection, and data acquisition. MD contributed experimental design analysis and interpretation of the data. TC contributed data acquisition, analysis and interpretation of the data. TN, KU, YK, TT, HH, and KNa contributed sample preparation, patient recruitment. MK contributed experimental design, sample preparation, study supervision. KNi contributed experimental design, sample preparation, writing the first draft of the paper. All authors contributed to the article and approved the submitted version.

## Funding

This work was supported in part by a Grant-in Aid for Scientific Research (C) from the Japan Society for the Promotion of Science Grant Numbers JP19K07722 awarded to KS and in part by a Grant-in Aid for Scientific Research on Innovative Areas “Frontier Research on Chemical Communications” (No JP17H06400 & JP17H06404) awarded to KN.

## Conflict of Interest

KS reports personal fees from Roche Diagnostics, Bio‐Rad, SRL Diagnostics, AstraZeneca, Chugai Pharmaceutical outside the submitted work. KU has received personal fees and honoraria from Eisai. HH reported grants from the Japan Agency for Medical Research and Development during the conduct of the study and grants and personal fees from AstraZeneca, Boehringer Ingelheim Japan Inc, Chugai Pharmaceutical, Ono Pharmaceutical, and Bristol-Myers Squibb and personal fees from Eli Lilly Japan, Kyorin Pharmaceutical, Merck Biopharma, MSD, Novartis, Pfizer Japan, Shanghai Haihe Biopharma, and Taiho Pharmaceutical outside the submitted work. KNa reports grants from Novartis, Boehringer Ingelheim, Pfizer, Takeda, SymBio Pharmaceuticals, Kyorin Pharmaceutical, CareNet, Nichi-Iko Pharmaceutical, Daiichi-Sankyo, Hisamitsu Pharmaceutical, Yodosha, Clinical Trial, Medicus Shuppan Publishers, Ayumi Pharmaceutical, Nikkei Business Publications, Thermo Fisher Scientific, Nanzando, Medical Review, Yomiuri Telecasting, Reno Medical, MSD, Eli Lilly, Bristol-Myers Squibb, Taiho Pharmaceutical, Ono Pharmaceutical, Chugai Pharmaceutical, AstraZeneca, Astellas, and grants from Novartis, Boehringer Ingelheim, Pfizer, Takeda, SymBio Pharmaceuticals, Daiichi-Sankyo, Merck Serono, ICON, Parexel International, IQVIA Services, A2 Healthcare, AbbVie, EP-CRSU, Linical, Otsuka Pharmaceutical, EPS, Quintiles, CMIC Shift Zero, Eisai, Kissei Pharmaceutical, Kyowa Hakko Kirin, Bayer, inVentiv Health, Gritstone Oncology, GlaxoSmithKline, Covance, MSD, Eli Lilly, Bristol-Myers Squibb, Taiho Pharmaceutical, Ono Pharmaceutical, Chugai Pharmaceutical, AstraZeneca, Astellas outside the submitted work. MK received fees for advisory role from Eisai, Ono, MSD, Bristol-Myers Squibb, and Roche, lecture fees from Eisai, Bayer, MSD, Bristol-Myers Squibb, Eli Lilly, and EA Pharma, and research funding from Gilead Sciences, Taiho, Sumitomo Dainippon Pharma, Takeda, Otsuka, EA Pharma, Abbvie, and Eisai. KNi reports personal fees from Otsuka Pharmaceutical, Life Technologies Japan, Boehringer Ingelheim, Eli Lilly, Chugai Pharmaceutical, Eisai, Pfizer, Novartis, MSD, Ono Pharmaceutical, Bristol‐Myers Squibb, SymBio Pharmaceuticals Limited, Solasia Pharma, Yakult Honsha, Roche Diagnostics, AstraZeneca, Sanofi, Guardant Health, Takeda, Kobayashi Pharmaceutical, and grants from Otsuka Pharmaceutical, Life Technologies Japan, Boehringer Ingelheim, Eli Lilly, Ignyta, Astellas outside the submitted work.

The remaining authors declare that the research was conducted in the absence of any commercial or financial relationships that could be construed as a potential conflict of interest.

## Publisher’s Note

All claims expressed in this article are solely those of the authors and do not necessarily represent those of their affiliated organizations, or those of the publisher, the editors and the reviewers. Any product that may be evaluated in this article, or claim that may be made by its manufacturer, is not guaranteed or endorsed by the publisher.

## Supplementary Material

Supplementary Figure 1 Relationships between irAE and UC in a pathway enrichment analysis. **(A)** Scatterplot of irAE and UC in inactive regions (Pearson correlation coefficient; r=0.5482, *p*=1.8528e-8). **(B)** Scatterplot of inactive and active regions of irAE (Pearson correlation coefficient; r=0.7112, *p*=1.3676e-15). **(C)** Scatterplot of irAE and UC in active regions (Pearson correlation coefficient; r=0.9581, *p*=1.6222e-159). **(D)** Scatterplot of inactive and active regions of UC (Pearson correlation coefficient; r=0.2212, *p*=0.0003).

Supplementary Figure 2 Similar regulatory mechanisms related to inflamed regions of irAE colitis and UC. **(A)** Hierarchical clustering using one minus Pearson correlation distance and average linkage for individual disease. **(B)** Genes in clusters 1 and 2. **(C)** Differential gene expression of upstream regulatory molecules (ICAM1, ITGB2, CD40, CD80, CD86, CD28, and CD4) associated with enhanced expression. Data are represented as the mean ± SD. *P* values were based on the Mann-Whitney U test. ***P* < 0.005; **P* < 0.05.

Supplementary Figure 3 Differential expression analysis between inflamed (active) regions of irAE colitis (A-irAE) and UC (A-UC). **(A)** Diagrams of the top 10 canonical pathways in an enrichment analysis between A-irAE colitis and A-UC. The bar plots show the z-score, which reflects the overall predicted activation state (<0: inhibited, >0: activated) (left), and the *p* value (−log) (right). **(B)** Ten most statistically significant upstream regulatory molecules in A-UC. The bar plots show the z-score, which reflects the overall predicted activation state (<0: inhibited, >0: activated) (left), and the *p* value (−log) (right).

Supplementary Table 1 Heatmap showing microbial taxa at the species level between inactive and active tissues.

Supplementary Table 2 Differentially abundant pathways.
