# Supplementary figures and images for: Intestinal Microbiota and Gene Expression Reveal Similarity and Dissimilarity Between Immune-Mediated Colitis and Ulcerative Colitis
Source: Front Oncol. 2021 Oct 27;11:763468. doi: 10.3389/fonc.2021.763468 (PMC8578892; doi:10.3389/fonc.2021.763468)

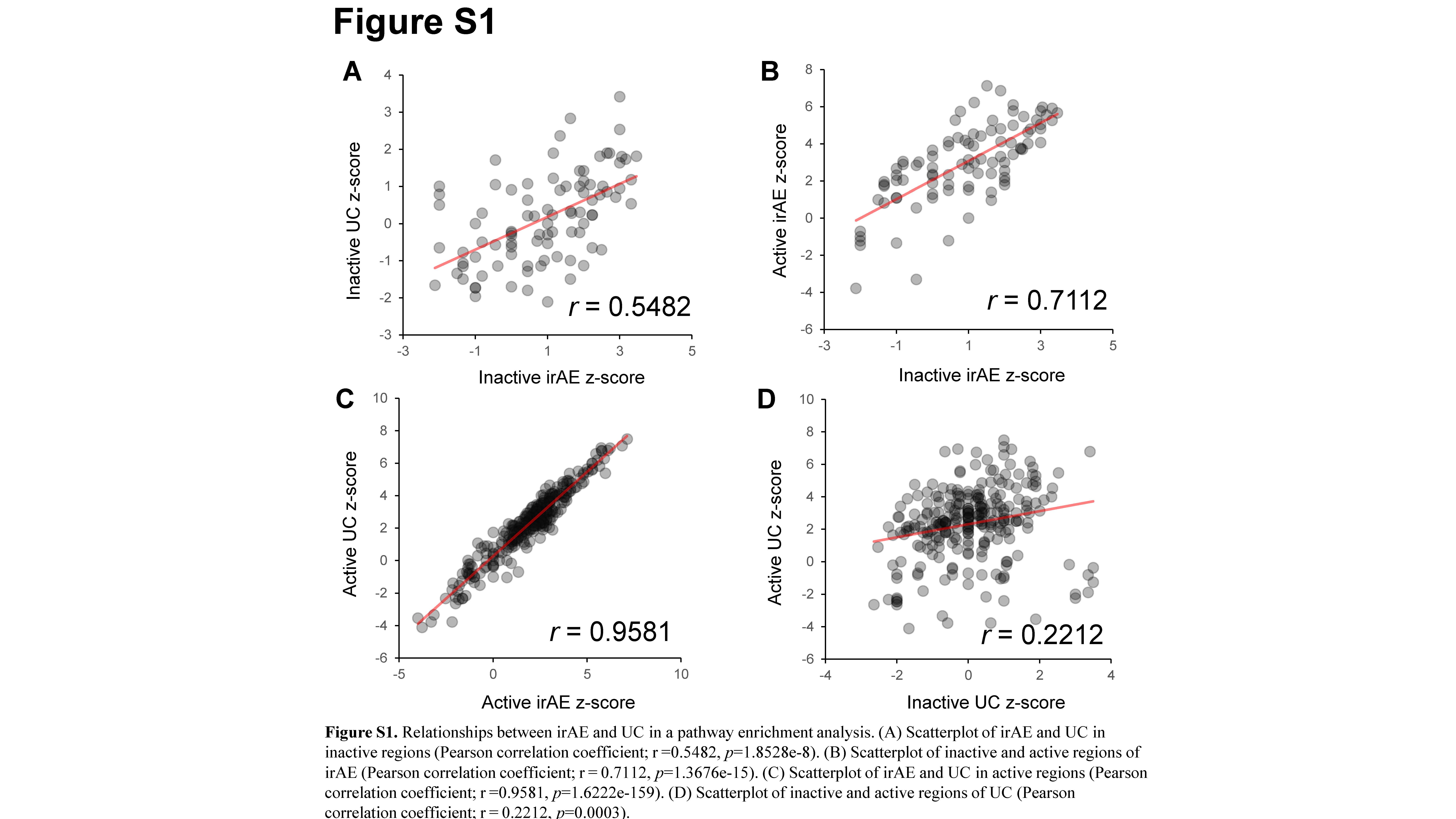

Supplement: Supplementary Figure 1 — Relationships between irAE and UC in a pathway enrichment analysis. (A) Scatterplot of irAE and UC in inactive regions (Pearson correlation coefficient; r=0.5482, p=1.8528e-8). (B) Scatterplot of inactive and active regions of irAE (Pearson correlation coefficient; r=0.7112, p=1.3676e-15). (C) Scatterplot of irAE and UC in active regions (Pearson correlation coefficient; r=0.9581, p=1.6222e-159). (D) Scatterplot of inactive and active regions of UC (Pearson correlation coefficient; r=0.2212, p=0.0003). [file Image_1.jpeg]

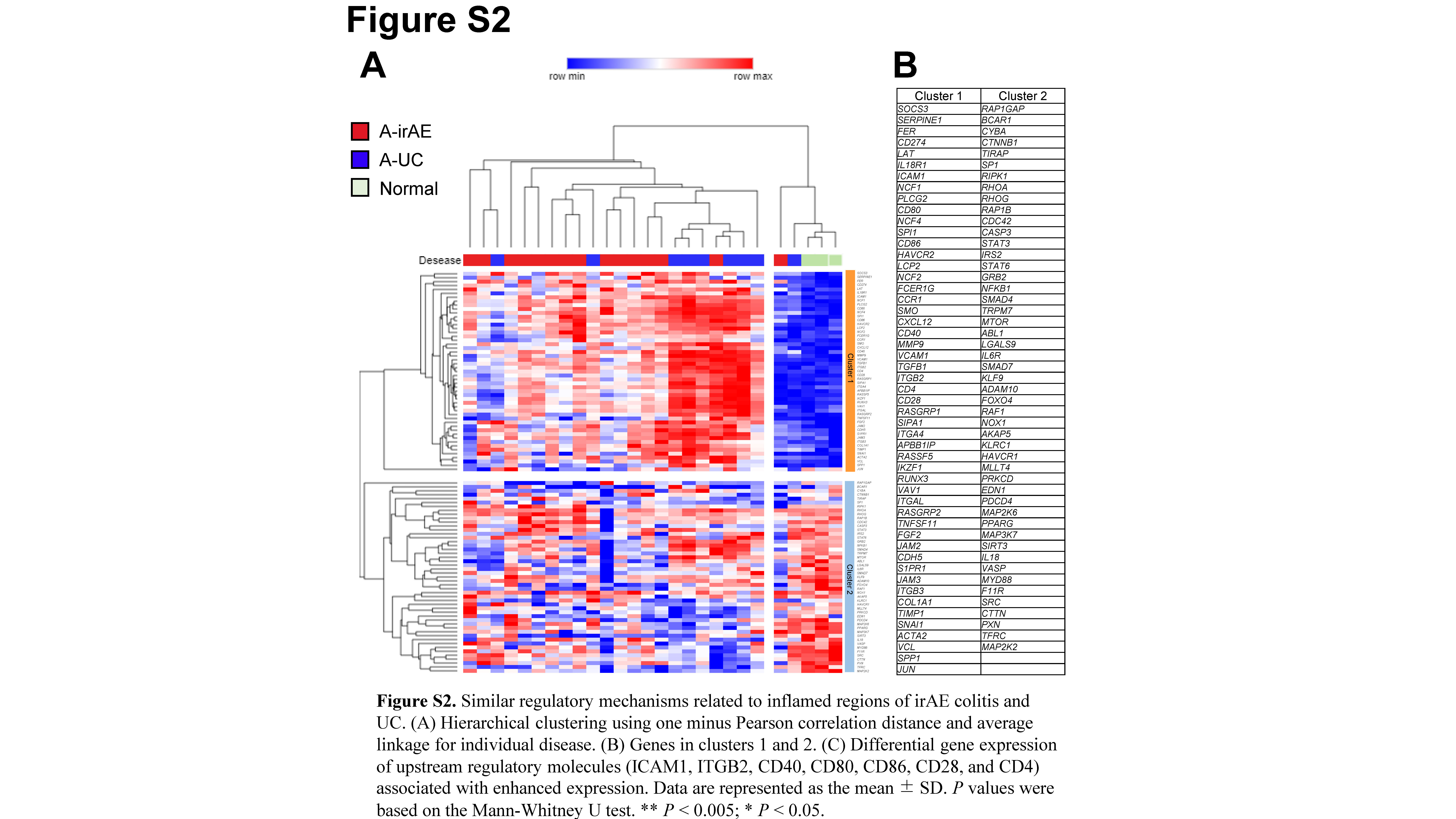

Supplement: Supplementary Figure 2 — Similar regulatory mechanisms related to inflamed regions of irAE colitis and UC. (A) Hierarchical clustering using one minus Pearson correlation distance and average linkage for individual disease. (B) Genes in clusters 1 and 2. (C) Differential gene expression of upstream regulatory molecules (ICAM1, ITGB2, CD40, CD80, CD86, CD28, and CD4) associated with enhanced expression. Data are represented as the mean ± SD. P values were based on the Mann-Whitney U test. **P < 0.005; *P < 0.05. [file Image_2.jpeg]

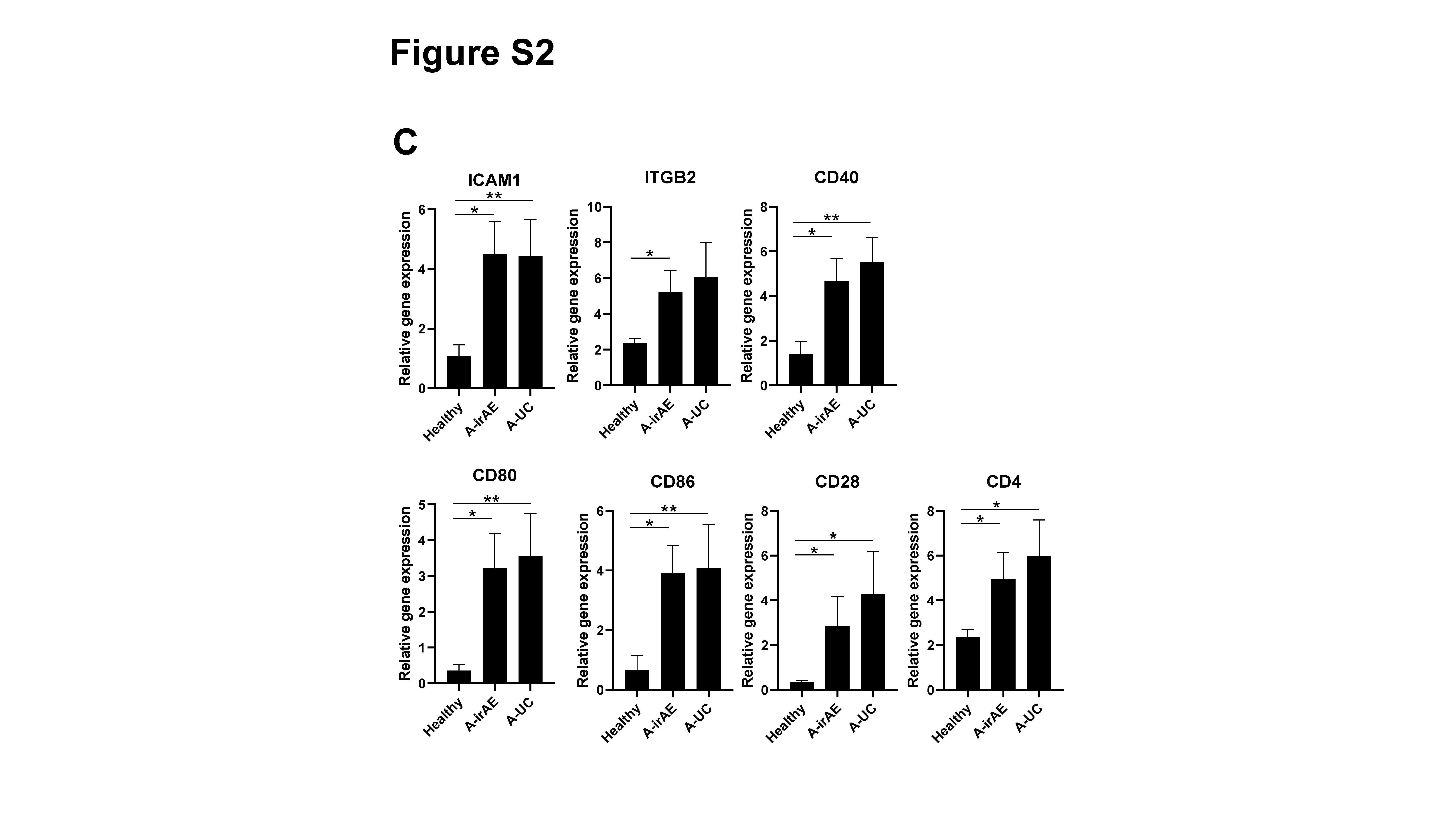

Supplement: Supplementary Figure 3 — Differential expression analysis between inflamed (active) regions of irAE colitis (A-irAE) and UC (A-UC). (A) Diagrams of the top 10 canonical pathways in an enrichment analysis between A-irAE colitis and A-UC. The bar plots show the z-score, which reflects the overall predicted activation state (<0: inhibited, >0: activated) (left), and the p value (−log) (right). (B) Ten most statistically significant upstream regulatory molecules in A-UC. The bar plots show the z-score, which reflects the overall predicted activation state (<0: inhibited, >0: activated) (left), and the p value (−log) (right). [file Image_3.jpeg]

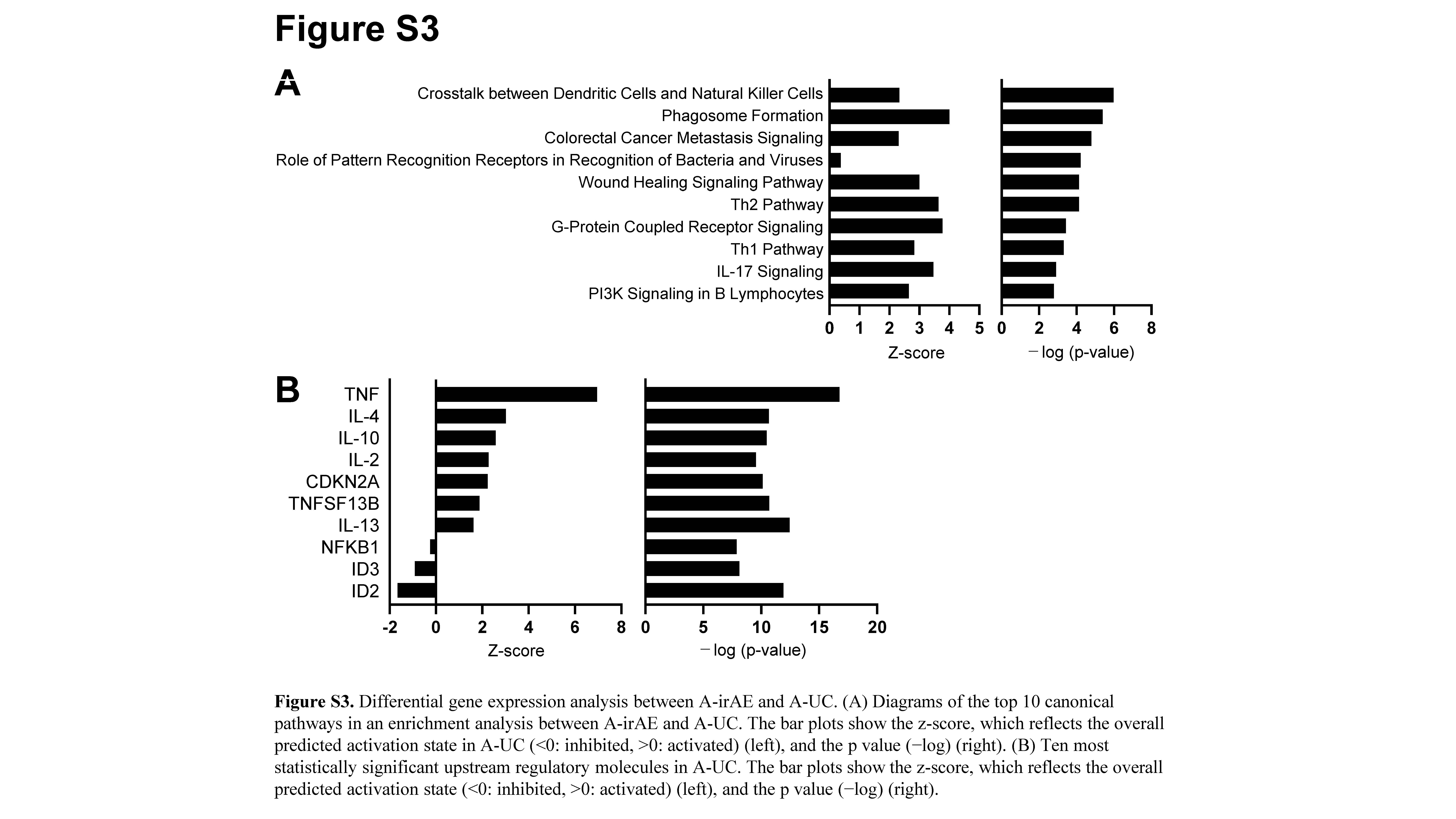

Supplement: Supplementary file 4 [file Image_4.jpeg]
