# Supplementary material for: Intestinal Microbiota and Gene Expression Reveal Similarity and Dissimilarity Between Immune-Mediated Colitis and Ulcerative Colitis
Source: Front Oncol. 2021 Oct 27;11:763468. doi: 10.3389/fonc.2021.763468 (PMC8578892; doi:10.3389/fonc.2021.763468)
Supplement: Supplementary Table 1 — Heatmap showing microbial taxa at the species level between inactive and active tissues. [file Table_1.docx]

**Table S1.** Heatmap showing microbial taxa at the species level between inactive and active tissues.

|  | Species-level taxonomy | Average abundance css (TSS) | | | | | *p*-value | | | | Average abundance css (TSS) | | *p*-value |
| --- | --- | --- | --- | --- | --- | --- | --- | --- | --- | --- | --- | --- | --- |
|  |  | I-irAE | I-UC | A-irAE | A-UC | Normal | I-irAE vs. Normal | I-UC vs. Normal | A-irAE vs. Normal | A-UC vs. Normal | Inactive | Active | Active vs. Inactive |
| Cluster 1 | Blautia_1104433 | 5.61 | 5.73 | 3.12 | 2.46 | 6.15 | 0.7676 | 0.7833 | 0.1373 | 0.0909 | 5.65 | 2.87 | **0.0328** |
| Cluster 1 | Unclassified.Lachnospiraceae_578032 | 3.45 | 6.59 | 1.95 | 2.44 | 4.15 | 0.5765 | 0.3833 | **<0.0001** | 0.3636 | 4.50 | 2.14 | **0.0340** |
| Cluster 1 | Faecalibacterium_prausnitzii__851865 | 4.71 | 6.17 | 0.98 | 0.89 | 3.65 | 0.6794 | 0.5167 | 0.7010 | 0.7636 | 5.20 | 0.94 | **0.0006** |
| Cluster 1 | Blautia_178736 | 5.05 | 5.47 | 0.33 | 0.49 | 5.65 | 0.7676 | 0.7833 | **0.0019** | **0.0074** | 5.19 | 0.39 | **<0.0001** |
| Cluster 1 | Bacteroides_ovatus__578688 | 4.55 | 3.55 | 3.36 | 1.74 | 4.60 | 0.9471 | 0.9333 | 0.7549 | 0.3182 | 4.22 | 2.75 | 0.3147 |
| Cluster 1 | Bacteroides_uniformis__800218 | 3.93 | 3.80 | 2.43 | 1.36 | 6.98 | 0.2265 | 0.3333 | 0.1373 | 0.2727 | 3.89 | 2.03 | 0.2348 |
| Cluster 1 | Odoribacter_210303 | 3.45 | 3.51 | 3.60 | 1.30 | 1.93 | 0.5765 | 0.6667 | 0.5490 | 0.7636 | 3.47 | 2.74 | 0.4530 |
| Cluster 1 | Streptococcus_1096637 | 4.91 | 4.79 | 1.43 | 1.65 | 3.07 | 0.4529 | 0.7333 | 0.2941 | 0.5091 | 4.87 | 1.51 | **0.0006** |
| Cluster 1 | Bacteroides_586379 | 5.97 | 9.14 | 0.92 | 0.28 | 10.93 | 0.0824 | 0.6667 | **0.0054** | **0.0042** | 7.03 | 0.68 | **<0.0001** |
| Cluster 1 | Phascolarctobacterium_587536 | 4.04 | 6.74 | 0.60 | 0.65 | 7.19 | 0.2353 | 0.7833 | **<0.0001** | **<0.0001** | 4.94 | 0.62 | **0.0003** |
| Cluster 1 | Helicobacter_206538 | 6.05 | 5.43 | 0.00 | 0.00 | 7.76 | 0.6794 | 0.5167 | **<0.0001** | **0.0016** | 5.84 | 0.00 | **<0.0001** |
| Cluster 1 | Bacteroides_uniformis__338710 | 3.88 | 6.08 | 1.01 | 0.63 | 5.77 | 0.4941 | 0.8333 | **<0.0001** | **<0.0001** | 4.61 | 0.87 | **0.0024** |
| Cluster 1 | Shigella_flexneri__676074 | 6.94 | 9.27 | 1.88 | 2.86 | 7.28 | 0.9706 | 0.1833 | **<0.0001** | **0.0160** | 7.72 | 2.25 | **<0.0001** |
| Cluster 1 | Escherichia_coli__783913 | 7.10 | 9.87 | 0.60 | 0.77 | 7.98 | 0.8265 | 0.1833 | **0.0019** | **0.0103** | 8.03 | 0.66 | **<0.0001** |
| Cluster 1 | Ruminococcus_gnavus__552961 | 6.50 | 7.25 | 2.26 | 4.67 | 9.24 | 0.4118 | 0.3833 | **<0.0001** | **0.0182** | 6.75 | 3.16 | **0.0025** |
| Cluster 1 | Ruminococcus_gnavus__2565881 | 5.62 | 6.76 | 0.61 | 1.12 | 8.49 | 0.3353 | 0.5167 | **0.0019** | **0.0127** | 6.00 | 0.80 | **<0.0001** |
| Cluster 1 | Bacteroides_562995 | 8.49 | 9.08 | 0.00 | 0.25 | 9.63 | 0.8588 | 0.8333 | **<0.0001** | **0.0042** | 8.68 | 0.09 | **<0.0001** |
| Cluster 1 | Clostridium_bolteae__851797 | 6.73 | 6.88 | 3.40 | 4.30 | 4.28 | 0.4294 | 0.3000 | 0.8578 | 0.9909 | 6.78 | 3.74 | **0.0240** |
| Cluster 1 | Haemophilus_parainfluenzae__997957 | 1.87 | 4.06 | 3.52 | 4.64 | 4.29 | 0.3529 | 1.0000 | 0.8676 | 0.8000 | 2.60 | 3.94 | 0.0954 |
| Cluster 1 | Fusobacterium_2078948 | 2.88 | 5.14 | 2.97 | 2.80 | 4.97 | 0.4588 | 0.9333 | 0.6569 | 0.7273 | 3.63 | 2.90 | 1.0000 |
| Cluster 1 | Bacteroides_fragilis__882886 | 8.27 | 3.56 | 5.98 | 4.74 | 4.69 | 0.2971 | 0.6333 | 0.9142 | 1.0000 | 6.70 | 5.52 | 0.6391 |
| Cluster 1 | Bacteroides_fragilis__3474081 | 7.74 | 3.47 | 1.78 | 1.29 | 4.25 | 0.3618 | 0.6667 | 0.2059 | **0.0364** | 6.32 | 1.60 | **0.0034** |
| Cluster 1 | Bacteroides_fragilis__577291 | 5.60 | 2.93 | 6.03 | 4.05 | 1.28 | 0.2353 | 0.5000 | 0.1176 | 0.3455 | 4.71 | 5.28 | 0.7397 |
| Cluster 2 | Bacteroides_841108 | 3.89 | 6.40 | 4.02 | 3.28 | 4.39 | 0.9059 | 0.6667 | 0.8578 | 0.6364 | 4.73 | 3.74 | 0.6069 |
| Cluster 2 | Parabacteroides_180082 | 4.08 | 5.34 | 3.20 | 2.06 | 5.18 | 0.9059 | 0.8333 | 0.7353 | 0.5091 | 4.50 | 2.77 | 0.2164 |
| Cluster 2 | Bacteroides_366744 | 5.04 | 3.98 | 3.09 | 2.68 | 2.97 | 0.5765 | 0.8333 | 0.8456 | 0.8636 | 4.68 | 2.94 | 0.2965 |
| Cluster 2 | Bacteroides_850870 | 5.06 | 5.18 | 6.36 | 4.70 | 9.74 | 0.0912 | 0.2667 | 0.3431 | 0.2000 | 5.10 | 5.73 | 0.5823 |
| Cluster 2 | Parabacteroides_distasonis__624618 | 4.14 | 5.03 | 5.63 | 2.89 | 4.89 | 0.9971 | 1.0000 | 0.9436 | 0.7273 | 4.44 | 4.60 | 0.8263 |
| Cluster 2 | Bacteroides_584695 | 1.23 | 2.19 | 5.03 | 4.03 | 3.47 | 0.3529 | 0.7000 | 0.4951 | 1.0000 | 1.55 | 4.65 | **0.0156** |
| Cluster 2 | Bacteroides_ovatus__844958 | 1.02 | 3.23 | 4.72 | 2.62 | 4.92 | **<0.0001** | 0.9333 | 0.7647 | 0.3182 | 1.76 | 3.93 | 0.1615 |
| Cluster 2 | Bacteroides_583117 | 1.88 | 5.53 | 2.53 | 3.19 | 3.73 | 0.3529 | 0.5667 | 0.4951 | 0.7273 | 3.10 | 2.78 | 0.9720 |
| Cluster 2 | Bacteroides_849273 | 2.64 | 2.92 | 3.29 | 1.54 | 7.12 | 0.1029 | 0.1667 | 0.0858 | **0.0144** | 2.74 | 2.63 | 0.8666 |
| Cluster 2 | Bacteroides_361962 | 7.35 | 7.52 | 7.79 | 6.53 | 11.87 | 0.2441 | 0.3333 | 0.2794 | 0.1818 | 7.41 | 7.32 | 0.8816 |
| Cluster 3 | Renibacterium_112057 | 0.00 | 0.00 | 6.53 | 7.15 | 0.00 | ND | ND | **0.0090** | **0.0155** | 0.00 | 6.77 | **<0.0001** |
| Cluster 3 | Pelomonas_puraquae__1111417 | 0.00 | 0.00 | 5.96 | 6.89 | 0.00 | ND | ND | **0.0090** | **0.0155** | 0.00 | 6.31 | **<0.0001** |
| Cluster 3 | Ralstonia_688522 | 0.00 | 0.00 | 6.21 | 7.32 | 0.00 | ND | ND | **0.0245** | **0.0155** | 0.00 | 6.63 | **<0.0001** |
| Cluster 3 | Microbacterium_562212 | 0.00 | 0.00 | 7.47 | 7.88 | 0.00 | ND | ND | **0.0090** | **0.0155** | 0.00 | 7.63 | **<0.0001** |
| Cluster 3 | Unclassified.Comamonadaceae_285352 | 0.00 | 0.00 | 6.50 | 6.99 | 0.00 | ND | ND | **0.0090** | **0.0155** | 0.00 | 6.68 | **<0.0001** |
| Cluster 3 | Sphingomonas_887292 | 0.00 | 0.00 | 5.56 | 6.37 | 0.00 | ND | ND | **0.0098** | **0.0155** | 0.00 | 5.86 | **<0.0001** |
| Cluster 3 | Microbacterium_895008 | 0.00 | 0.00 | 5.49 | 5.84 | 0.00 | ND | ND | **0.0098** | **0.0364** | 0.00 | 5.63 | **<0.0001** |
| Cluster 3 | Cupriavidus_551264 | 0.00 | 0.00 | 9.49 | 10.13 | 0.00 | ND | ND | **0.0090** | **0.0155** | 0.00 | 9.73 | **<0.0001** |
| Cluster 3 | Microbacterium_306103 | 0.00 | 0.00 | 7.97 | 8.81 | 0.00 | ND | ND | **0.0098** | **0.0155** | 0.00 | 8.28 | **<0.0001** |
| Cluster 3 | Bacteroides_588308 | 0.00 | 0.00 | 8.42 | 6.59 | 0.00 | ND | ND | **0.0245** | 0.0909 | 0.00 | 7.73 | **<0.0001** |
| Cluster 3 | Bacteroides_589277 | 0.25 | 0.33 | 7.74 | 5.80 | 1.10 | **<0.0001** | 0.9333 | **0.0245** | 0.1455 | 0.27 | 7.02 | **<0.0001** |
| Cluster 3 | Unclassified | 4.43 | 5.83 | 10.87 | 10.93 | 4.48 | 0.5706 | 0.0667 | **0.0025** | **0.0091** | 4.90 | 10.89 | **<0.0001** |
| Cluster 3 | Unclassified.Enterobacteriaceae_1107949 | 0.42 | 0.87 | 6.46 | 10.08 | 0.00 | 0.6618 | 0.9333 | **0.0245** | **0.0155** | 0.57 | 7.82 | **<0.0001** |
| Cluster 3 | Unclassified.Enterobacteriaceae_1111141 | 0.00 | 0.00 | 5.97 | 9.23 | 0.00 | ND | ND | **0.0490** | **0.0155** | 0.00 | 7.19 | **<0.0001** |
| Cluster 3 | Unclassified.Enterobacteriaceae_1111717 | 0.00 | 0.00 | 7.71 | 9.29 | 0.00 | ND | ND | **0.0090** | **0.0364** | 0.00 | 8.30 | **<0.0001** |
| Cluster 3 | Shigella_flexneri__4349524 | 0.92 | 0.77 | 4.77 | 7.10 | 0.00 | 0.9294 | 0.9333 | 0.0858 | **0.0155** | 0.87 | 5.64 | **<0.0001** |
| Cluster 3 | Klebsiella_1061273 | 0.00 | 0.00 | 5.97 | 5.62 | 0.00 | ND | ND | **0.0245** | **0.0364** | 0.00 | 5.84 | **<0.0001** |
