# Supplementary material for: Intestinal Microbiota and Gene Expression Reveal Similarity and Dissimilarity Between Immune-Mediated Colitis and Ulcerative Colitis
Source: Front Oncol. 2021 Oct 27;11:763468. doi: 10.3389/fonc.2021.763468 (PMC8578892; doi:10.3389/fonc.2021.763468)
Supplement: Supplementary Table 2 — Differentially abundant pathways. [file Table_2.docx]

**Table S2.** Differentially abundant pathways

| KEGG pathway | | Median abundance (range) | | | | | Fold change over the normal group (vs normal) | | | | *p*-value (vs normal) | | | |
| --- | --- | --- | --- | --- | --- | --- | --- | --- | --- | --- | --- | --- | --- | --- |
|  |  | I-irAE | I-UC | A-irAE | A-UC | Normal | I-irAE | I-UC | A-irAE | A-UC | I-irAE | I-UC | A-irAE | A-UC |
| ko02020 | Two-component system | 216065 (88971 - 1078912) | 277027 (88971 - 1626072) | 759076 (94968 - 1113013) | 586213 (213497 - 927743) | 131824 (127361 - 163596) | 2.6944 | 3.2274 | 4.4266 | 4.3236 | 0.0324 | 0.3833 | 0.0172 | 0.0091 |
| ko02010 | ABC transporters | 372975 (141720 - 1691487) | 427212 (141720 - 2696570) | 1057216 (144640 - 1523754) | 831398 (325065 - 1330573) | 198201 (196216 - 245575) | 2.6771 | 3.3698 | 4.2081 | 4.0643 | 0.0471 | 0.8333 | 0.0172 | 0.0091 |
| ko02024 | Quorum sensing | 202295 (76255 - 798708) | 225016 (76255 - 1606427) | 467154 (89404 - 876520) | 366542 (233961 - 960806) | 110505 (98380 - 121285) | 2.8261 | 3.8954 | 4.1223 | 4.3880 | 0.0118 | 0.3833 | 0.0172 | 0.0091 |
| ko00650 | Butanoate metabolism | 79862 (26879 - 308112) | 87458 (26879 - 861443) | 189515 (48566 - 340632) | 164304 (121586 - 354848) | 47256 (42540 - 54729) | 2.3528 | 4.2232 | 4.0749 | 4.4433 | 0.0471 | 0.1833 | 0.0049 | 0.0091 |
| ko03070 | Bacterial secretion system | 88397 (22849 - 286486) | 90695 (22849 - 785408) | 221033 (46967 - 321806) | 187407 (102417 - 289677) | 43430 (42324 - 54607) | 2.4423 | 4.1083 | 4.0348 | 4.1862 | 0.0118 | 0.1833 | 0.0049 | 0.0091 |
| ko00640 | Propanoate metabolism | 93292 (27630 - 341756) | 109697 (27630 - 1276826) | 187281 (60960 - 338713) | 199428 (99257 - 349112) | 56804 (47019 - 63988) | 2.3652 | 4.8409 | 3.7218 | 3.9986 | 0.0471 | 0.1833 | 0.0049 | 0.0091 |
| ko01212 | Fatty acid metabolism | 78354 (23391 - 319593) | 92285 (23391 - 859107) | 148131 (56513 - 328990) | 179208 (128090 - 340927) | 50288 (35484 - 61589) | 2.2099 | 4.1096 | 3.5463 | 4.0527 | 0.0471 | 0.2667 | 0.0049 | 0.0091 |
| ko01120 | Microbial metabolism in diverse environments | 636985 (212724 - 2347276) | 772937 (212724 - 8527457) | 1379484 (435275 - 2198059) | 1416697 (887135 - 2309965) | 416003 (343448 - 494349) | 2.2348 | 4.5531 | 3.4108 | 3.7010 | 0.0471 | 0.2667 | 0.0049 | 0.0091 |
| ko00564 | Glycerophospholipid metabolism | 70539 (23643 - 244988) | 86438 (23643 - 908070) | 147509 (36991 - 218449) | 135310 (92030 - 190262) | 42750 (35404 - 46225) | 2.3687 | 4.8524 | 3.3908 | 3.4327 | 0.0118 | 0.1167 | 0.0098 | 0.0091 |
| ko00790 | Folate biosynthesis | 78495 (21398 - 220530) | 92711 (21398 - 973177) | 145154 (50742 - 240161) | 150776 (122798 - 219410) | 43697 (32849 - 56690) | 2.2035 | 4.9108 | 3.2950 | 3.6088 | 0.0471 | 0.1833 | 0.0049 | 0.0091 |
| ko00620 | Pyruvate metabolism | 152871 (48180 - 433371) | 179478 (48180 - 1945273) | 296510 (103131 - 471892) | 321502 (214206 - 469294) | 93369 (73865 - 116911) | 2.1186 | 4.5671 | 3.2389 | 3.4923 | 0.0324 | 0.2667 | 0.0049 | 0.0091 |
| ko00860 | Porphyrin and chlorophyll metabolism | 97583 (30903 - 317276) | 103715 (30903 - 506223) | 187264 (54183 - 280380) | 179394 (71807 - 226659) | 50546 (39791 - 67102) | 2.3522 | 3.0089 | 3.1824 | 3.0636 | 0.0471 | 0.2667 | 0.0049 | 0.0091 |
| ko01200 | Carbon metabolism | 376688 (118150 - 1121872) | 449178 (118150 - 5131067) | 728656 (257983 - 1100381) | 806218 (566886 - 1110535) | 241070 (163111 - 294769) | 2.1508 | 4.8497 | 3.1078 | 3.4139 | 0.0471 | 0.1833 | 0.0049 | 0.0091 |
| ko00230 | Purine metabolism | 273788 (88304 - 816516) | 326677 (88304 - 3470429) | 563740 (180408 - 792789) | 565860 (415928 - 706515) | 171639 (118567 - 217868) | 2.1926 | 4.6378 | 3.0213 | 3.2372 | 0.0324 | 0.2667 | 0.0049 | 0.0091 |
| ko00270 | Cysteine and methionine metabolism | 122691 (43366 - 429588) | 159195 (43366 - 1675952) | 260443 (77376 - 370425) | 259750 (186884 - 317045) | 79783 (57096 - 96323) | 2.2379 | 4.8288 | 2.9846 | 3.2063 | 0.0324 | 0.1833 | 0.0098 | 0.0091 |
| ko00770 | Pantothenate and CoA biosynthesis | 71322 (24424 - 247139) | 91418 (24424 - 858865) | 154394 (45355 - 210063) | 156499 (91731 - 198216) | 45790 (34074 - 58195) | 2.3193 | 4.4919 | 2.9727 | 3.1653 | 0.0471 | 0.1833 | 0.0098 | 0.0091 |
| ko00720 | Carbon fixation pathways in prokaryotes | 127020 (38952 - 341589) | 152399 (38952 - 1844452) | 222969 (92324 - 339599) | 239385 (172057 - 327959) | 79795 (49128 - 104458) | 2.0561 | 5.1536 | 2.8488 | 3.1815 | 0.0324 | 0.1833 | 0.0049 | 0.0091 |
| ko00240 | Pyrimidine metabolism | 216971 (69448 - 630526) | 262263 (69448 - 2807718) | 402245 (146457 - 587996) | 411303 (308938 - 503253) | 136623 (90781 - 175830) | 2.1045 | 4.7003 | 2.8449 | 3.0781 | 0.0324 | 0.1833 | 0.0049 | 0.0091 |
| ko00550 | Peptidoglycan biosynthesis | 87913 (32690 - 318805) | 113400 (32690 - 1102422) | 158283 (53964 - 223421) | 175553 (114752 - 200361) | 56780 (37676 - 68153) | 2.2235 | 4.6463 | 2.7873 | 3.0617 | 0.0324 | 0.1833 | 0.0098 | 0.0091 |
| ko03030 | DNA replication | 77795 (24624 - 235732) | 93774 (24624 - 1008382) | 133278 (48056 - 181347) | 138849 (92833 - 167511) | 46248 (28839 - 59522) | 2.2898 | 5.0519 | 2.7694 | 3.0508 | 0.0324 | 0.1833 | 0.0049 | 0.0091 |
| ko03060 | Protein export | 77174 (24392 - 231428) | 94559 (24392 - 888561) | 135060 (49500 - 187412) | 141328 (98082 - 174521) | 48386 (29018 - 61071) | 2.2232 | 4.5616 | 2.7569 | 2.9164 | 0.0471 | 0.1833 | 0.0049 | 0.0091 |
| ko03430 | Mismatch repair | 93337 (31384 - 326067) | 117104 (31384 - 1192614) | 161137 (57381 - 227430) | 165319 (118253 - 197466) | 58868 (36746 - 71774) | 2.2306 | 4.8262 | 2.7391 | 2.9836 | 0.0324 | 0.1833 | 0.0098 | 0.0091 |
| ko03440 | Homologous recombination | 105747 (34129 - 348295) | 131267 (34129 - 1372000) | 191025 (70293 - 266722) | 193599 (139063 - 232612) | 68156 (42196 - 87383) | 2.1620 | 4.7565 | 2.7121 | 2.9624 | 0.0471 | 0.1833 | 0.0049 | 0.0091 |
| ko00190 | Oxidative phosphorylation | 150521 (39032 - 545577) | 185282 (39032 - 1963365) | 252286 (101532 - 431205) | 300012 (216851 - 438873) | 89190 (63380 - 120171) | 2.3168 | 4.9103 | 3.0871 | 3.4287 | 0.0676 | 0.1833 | 0.0049 | 0.0091 |
| ko01210 | 2-Oxocarboxylic acid metabolism | 99512 (36266 - 369908) | 135518 (36266 - 1345872) | 200492 (68613 - 290189) | 227690 (81213 - 298078) | 69227 (45751 - 88128) | 2.2070 | 4.6165 | 2.8818 | 3.0596 | 0.0676 | 0.2667 | 0.0098 | 0.0182 |
| ko00220 | Arginine biosynthesis | 65463 (23115 - 230637) | 86419 (23115 - 887990) | 133813 (48417 - 192507) | 142626 (73477 - 177821) | 45990 (27797 - 59837) | 2.1742 | 4.5823 | 2.8147 | 2.9583 | 0.0676 | 0.2667 | 0.0049 | 0.0091 |
| ko00400 | Phenylalanine, tyrosine and tryptophan biosynthesis | 92077 (31523 - 345270) | 123999 (31523 - 1239966) | 171450 (62869 - 236488) | 170193 (119525 - 222526) | 62655 (39406 - 81790) | 2.1671 | 4.7468 | 2.5813 | 2.8133 | 0.0676 | 0.1833 | 0.0049 | 0.0091 |
| ko01110 | Biosynthesis of secondary metabolites | 1026263 (328128 - 3192953) | 1283320 (328128 - 14182307) | 2101913 (696681 - 2927261) | 2233575 (1536845 - 2809335) | 671896 (446921 - 846953) | 2.1661 | 4.8386 | 2.9539 | 3.1972 | 0.0676 | 0.1833 | 0.0049 | 0.0091 |
| ko03010 | Ribosome | 320679 (104002 - 852444) | 380297 (104002 - 4007919) | 538601 (213002 - 755733) | 550215 (395166 - 660244) | 195041 (118558 - 263183) | 2.1229 | 4.8115 | 2.6554 | 2.8698 | 0.0676 | 0.1833 | 0.0049 | 0.0091 |
| ko01230 | Biosynthesis of amino acids | 494741 (180287 - 1789504) | 669658 (180287 - 7024840) | 986917 (356448 - 1347543) | 984898 (644034 - 1281458) | 349779 (212013 - 438538) | 2.1191 | 4.7636 | 2.6864 | 2.9115 | 0.0676 | 0.2667 | 0.0049 | 0.0091 |
| ko01100 | Metabolic pathways | 2395332 (754123 - 6968517) | 2962571 (754123 - 33445811) | 4928009 (1700294 - 7051819) | 5229906 (3584486 - 6551497) | 1590033 (1064892 - 1990396) | 2.1120 | 4.7716 | 2.9354 | 3.1559 | 0.0676 | 0.2667 | 0.0049 | 0.0091 |
| ko00260 | Glycine, serine and threonine metabolism | 112733 (35398 - 355977) | 138582 (35398 - 1641099) | 221483 (81398 - 351063) | 249681 (177562 - 339843) | 74136 (53817 - 95121) | 2.1044 | 4.8192 | 3.0634 | 3.3676 | 0.0676 | 0.2667 | 0.0049 | 0.0091 |
| ko00710 | Carbon fixation in photosynthetic organisms | 74708 (24993 - 220943) | 95943 (24993 - 979059) | 142625 (53894 - 216865) | 145744 (104886 - 194044) | 52466 (32402 - 61530) | 2.0832 | 4.5842 | 2.7832 | 3.0475 | 0.0676 | 0.2667 | 0.0049 | 0.0091 |
| ko00970 | Aminoacyl-tRNA biosynthesis | 368455 (117138 - 1376022) | 476851 (117138 - 3746242) | 703849 (282532 - 931927) | 708224 (534219 - 899220) | 242856 (144923 - 355049) | 2.0823 | 3.9629 | 2.6995 | 2.9028 | 0.0676 | 0.1833 | 0.0049 | 0.0091 |
| ko00061 | Fatty acid biosynthesis | 71366 (21344 - 228839) | 87978 (21344 - 854409) | 123313 (54878 - 193236) | 134027 (95172 - 199335) | 49032 (28090 - 60616) | 1.9989 | 4.2701 | 2.8047 | 3.1473 | 0.0676 | 0.2667 | 0.0049 | 0.0091 |
| ko00630 | Glyoxylate and dicarboxylate metabolism | 105986 (29109 - 458793) | 126742 (29109 - 1664864) | 205899 (78552 - 420214) | 232745 (160421 - 433221) | 69619 (46117 - 89428) | 2.2738 | 5.1856 | 3.4182 | 3.8330 | 0.0912 | 0.2667 | 0.0049 | 0.0091 |
| ko00680 | Methane metabolism | 97836 (32412 - 378221) | 123905 (32412 - 1321054) | 193183 (72379 - 287947) | 217921 (144338 - 286187) | 68143 (42388 - 80591) | 2.1447 | 4.6461 | 2.9484 | 3.2601 | 0.0912 | 0.1833 | 0.0049 | 0.0091 |
| ko01130 | Biosynthesis of antibiotics | 782470 (252985 - 2437575) | 998596 (252985 - 11277045) | 1546671 (573713 - 2215791) | 1686044 (1140888 - 2209723) | 537770 (337145 - 681176) | 2.1181 | 4.8278 | 2.8634 | 3.1369 | 0.0912 | 0.2667 | 0.0049 | 0.0091 |
| ko00020 | Citrate cycle (TCA cycle) | 93862 (24622 - 241860) | 108252 (24622 - 1402141) | 176710 (68110 - 268996) | 185079 (139372 - 261976) | 57688 (39594 - 78561) | 1.9502 | 5.1561 | 2.9706 | 3.3261 | 0.0912 | 0.1833 | 0.0049 | 0.0091 |
| ko00520 | Amino sugar and nucleotide sugar metabolism | 208634 (66926 - 632369) | 273579 (66926 - 3332881) | 332574 (165559 - 589074) | 389040 (269692 - 479925) | 151349 (80855 - 188520) | 1.9355 | 5.0057 | 2.5858 | 2.6649 | 0.0912 | 0.3833 | 0.0049 | 0.0091 |
| ko00500 | Starch and sucrose metabolism | 168581 (43200 - 750448) | 206301 (43200 - 2402519) | 258662 (91359 - 472223) | 277884 (179545 - 418294) | 110036 (76367 - 110803) | 2.1240 | 4.9199 | 2.8326 | 2.7812 | 0.1206 | 0.3833 | 0.0098 | 0.0091 |
| ko03018 | RNA degradation | 66915 (20496 - 195388) | 84022 (20496 - 985296) | 131987 (52240 - 177044) | 124465 (87583 - 160246) | 46403 (26355 - 62810) | 2.0703 | 4.9048 | 2.6004 | 2.8185 | 0.1206 | 0.1833 | 0.0049 | 0.0091 |
| ko00010 | Glycolysis / Gluconeogenesis | 164092 (55108 - 467323) | 197090 (55108 - 2329810) | 322442 (117690 - 505015) | 337094 (248914 - 435020) | 109546 (75561 - 132738) | 1.9871 | 4.7845 | 2.9180 | 3.1699 | 0.1206 | 0.2667 | 0.0049 | 0.0091 |
| ko00051 | Fructose and mannose metabolism | 124434 (48586 - 329207) | 163722 (48586 - 1692780) | 236366 (98523 - 446370) | 254202 (166684 - 416801) | 94855 (65769 - 103030) | 1.8465 | 4.2904 | 2.7642 | 3.0300 | 0.1206 | 0.6667 | 0.0049 | 0.0091 |
| ko00030 | Pentose phosphate pathway | 106379 (39399 - 372760) | 141703 (39399 - 1534354) | 224222 (82945 - 371166) | 232206 (130150 - 302865) | 81943 (54855 - 90079) | 1.9936 | 4.4794 | 2.8646 | 3.0117 | 0.1559 | 0.5167 | 0.0049 | 0.0091 |
| ko00670 | One carbon pool by folate | 61225 (19369 - 173614) | 80581 (19369 - 989207) | 112768 (48695 - 158866) | 116850 (76271 - 142855) | 44060 (23709 - 57580) | 1.9930 | 5.0785 | 2.5320 | 2.8209 | 0.1559 | 0.3833 | 0.0049 | 0.0091 |
| ko01501 | beta-Lactam resistance | 62314 (16436 - 172162) | 72978 (16436 - 939715) | 119316 (47290 - 191654) | 121735 (96011 - 153748) | 40616 (28776 - 54382) | 1.9454 | 4.8877 | 2.8312 | 2.9663 | 0.1559 | 0.3833 | 0.0049 | 0.0091 |
| ko00250 | Alanine, aspartate and glutamate metabolism | 121560 (42097 - 372755) | 162384 (42097 - 2030564) | 238370 (105869 - 347207) | 239680 (162340 - 303945) | 91930 (47522 - 120447) | 1.8994 | 4.9754 | 2.5790 | 2.7704 | 0.2441 | 0.3833 | 0.0049 | 0.0091 |
| ko00052 | Galactose metabolism | 128343 (42146 - 477122) | 183982 (42146 - 2864819) | 197441 (125031 - 360064) | 255381 (125584 - 350139) | 118020 (49485 - 130648) | 1.7048 | 5.6199 | 2.1661 | 2.2798 | 0.3000 | 0.3833 | 0.0172 | 0.0182 |
| ko00040 | Pentose and glucuronate interconversions | 59336 (16490 - 211689) | 92896 (16490 - 1322858) | 126265 (59160 - 240372) | 159753 (43568 - 205664) | 59020 (36661 - 60632) | 1.6402 | 5.0041 | 2.6781 | 2.7783 | 0.5912 | 0.3833 | 0.0049 | 0.0364 |
